# Preliminary study on the tick population of Benin wildlife at the moment of its invasion by the *Rhipicephalus microplus* tick (Canestrini, 1888)

**DOI:** 10.14202/vetworld.2018.845-851

**Published:** 2018-06-25

**Authors:** Kossi Justin Adinci, Yao Akpo, Camus Adoligbe, Safiou Bienvenu Adehan, Roland Eric Yessinou, Akoeugnigan Idelphonse Sodé, Guy Appolinaire Mensah, Abdou Karim Issaka Youssao, Brice Sinsin, Souaïbou Farougou

**Affiliations:** 1 Laboratory of Research in Applied Biology, Polytechnic School of Abomey-Calavi, University of Abomey-Calavi, 01 P.O. Box 2009, Cotonou, Benin; 2 Laboratory of Ecology, Health and Animal Production, Faculty of Agronomy, University of Parakou, P.O. Box 123 Parakou, Benin; 3 National Institute for Scientific Research, Research Center of Agonkanmey (CRA/INRAB), Benin; 4 Laboratory of Biomathematics and Forest Estimations Faculty of Agronomic Sciences (FSA) University of Abomey-Calavi, 04 BP 1525, Cotonou (Bénin); 5 Department of Animal Production, Faculty of Agronomic Sciences (FSA), University of Abomey-Calavi (Benin), 01 BP 526 Cotonou, Benin

**Keywords:** Benin, *Rhipicephalus microplus*, ticks, wild animals

## Abstract

**Background and Aim:**

*Rhipicephalus microplus* (*Rm*) is one of the most problematic livestock tick species in the world. Its rapid propagation and resistance to acaricides make it control difficult in the sub-region and Benin particularly. The aim of this work was to check its presence in wildlife and to confirm the possible role of reservoir wildlife may play in the propagation of the parasite. This will help to design more efficient control strategy.

**Materials and Methods:**

This study was conducted from February to March 2017 in the National Parks of Benin (Pendjari and W Park) and wildfowl’s assembly and selling point in Benin. Ticks were manually picked with forceps from each animal after slaughtering by hunters then stored in 70° ethanol. Collected ticks were counted and identified in the laboratory using the identification key as described by Walker.

**Results:**

Overall, seven species of ticks (*Amblyomma variegatum, Boophilus decoloratus, Rm, Boophilus* spp*., Hyalomma* spp*., Rhipicephalus sanguineus, Rhipicephalus* spp.) were identified on nine wild animal species sampled (Cane rat, wildcat, Hare, Doe, Cricetoma, Buffalo, Buffon Cobe, and Bushbuck and Warthog). The average number of ticks varies from 3 to 6 between animal species, 3 to 7 between localities visited, and 2 to 5 between tick species. However, these differences are statistically significant only for localities. Considering tick species and animal species, the parasite load of *Rm* and *Rhipicephalus* spp. is higher; the buffalo being more infested. The analysis of deviance reveals that the abundance of ticks observed depends only on the observed localities (p>0.05). However, the interactions between animal species and localities on the one hand and between animal and tick species on the other hand, although not significant, have influenced the abundance of ticks as they reduce the residual deviance after their inclusion in the model.

**Conclusions:**

This study reported the presence of *Rm* in wildlife of Benin and confirmed its role in the maintenance and spread of the parasites. It is, therefore, an important risk factor that we must not neglect in the epidemiological surveillance and ticks control strategies in the West African sub-region and particularly in Benin.

## Introduction

Ticks’ host, like all parasites host, plays an important role in their distribution. Ticks spend almost all their time with the host and move from one point to another with them. Female ticks leave their host and fall into the environment, lay eggs when they are fully engorged. New larvae will look for another host, and the cycle will restart [1]. Tick-borne diseases have a significant impact on animal productivity and cause economic losses for livestock owners. This is a major obstacle for the livestock sector development in Africa and Benin, in particular, due to the presence of a large number of tick species including *Rhipicephalus mipcroplus* (*Rm*), one of the most feared species [2]. The previous study has shown that *Rm* is a vector of *Babesia bovis, Babesia bigemina*, and *Anaplasma marginale* [3]. In Benin, the introduction of *Rm* was largely attributed to the importation of Girolando cattle from Brazil by the Government of Benin through the Pafilav Project which aim was to improve local breed milk production [4]. The first study conducted on October 2008 in the village of Kpinnou, the main site of Girolando cattle in Benin, indicated the presence of *Rm* and the suitability of local conditions for its development [5]. As stated by different findings, this tick species has rapidly spread all over the country [6]. Ticks collection and identification from domestic animals were done several times for research purposes.

Wildlife is often pointed to act as a reservoir of tick-borne disease for domestic animals and, *vice versa* [7-9]. Some studies confirm this perception for some diseases (severe acute respiratory syndrome, bird flu) [10-12]. For pastoralist, wildlife can be the cause of economic disasters when the survival or profitability of the domestic herd is threatened by epizootics or endozooties in which the fauna acts as a carrier, reservoir, or intermediate host [13]. However, no investigation has yet been done on wild species following the identification and spread of *Rm*, which stands out for its resistance to common acaricides and is a real problem for ruminant farms in Benin [14].

Studies related to the identification of the tick population of Benin’s wildlife are, therefore, necessary. These will make it possible to check the presence of this new species tick in the wildlife of Benin. Furthermore, they will allow to have a good knowledge of the acarological environment of this tick and to take it to account in the strategies of control.

## Materials and Methods

### Ethical approval

The samples taken during the present study knew no ethical requirements. In fact, these samples were taken from animals slaughtered by legal and illegal hunters.

### Study area

This study was conducted in Southern, Central, and Northern Benin located in the intertropical zone between parallels 6 ° 30 ‘ and 12 ° 30’ of north latitude on the one hand and meridians 1° and 3° 40’ of east longitude, on the other hand, Benin covers an area of 112,622 km^2^. The relief is slightly uneven, consisting of 2 plains and plateaus whose average altitude does not exceed 200 meters. The highest region (Atacora) where many rivers take their source from (Alibori, Mekrou…) is located at the northwestern part of the country (Alibori and Mekrou) Benin has three main climatic zones as follow:

- The northern part is characterized by a semi-arid Sudanese climate beyond latitude 10° N with a unimodal climatic regime (900–1100 mm of rain), two seasons (one dry and one rainy) and a beginning of saheli station, with shallow soils, often degraded and not very fertile;
- The central part own a transitional Sudano-Guinean climate, between 7° and 10° N parallels, with both unimodal and bimodal climatic conditions (1000-1200 mm of rain). It has poor colluvial soil at the reliefs foot and the top of the undulations, with a weak ecological situation in certain localities;
- The Southern part is characterized by a sub-equatorial climate (between parallels 6° 30’ and 7° N). It has four-seasons (two rainy and two dry seasons) with fertile soils and degradation of ecological conditions. The rainfall reaches 1500 mm.

Benin has five agro-pastoral zones: the dry Sudanese zone with marginal pastures, the Sudanese surplus grassland zone, the Sudano-Guinean zone with abundant forage resources, the semi-humid zone with agricultural vocation which becomes a zone of breeding and the forest zone [15,16]. Benin cattle herd is estimated at 2,166,000 head on 2015 [17], 90% of the animal is found in the Northern part of the country. The town of Banikoara, where W Park is located has the greatest number of cattle all over the country. The main ruminant breeds raised in these different agro-pastoral areas are Borgou, Somba, Ndama, Lagunaire, Mbororo, and White Fulani and their crossbred’s products for cattle; Djallonke sheep, Peulh sheep, Guinean dwarf goat, Maradi red goat, and Peulh goat for small ruminants. The whole country is suitable for animal husbandry, except a small area in the northern and southern part of the country [18].

### Sampling sites

**Figure-1:**
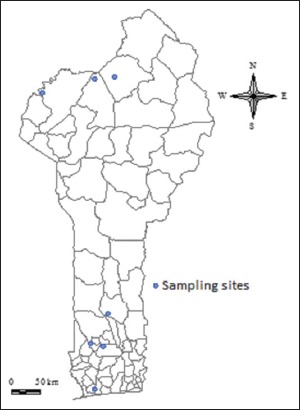
Sampling sites.

In southern and central Benin, samples were taken at the grouping and selling points of wildfowl in the commune of Kpomasse, Dassa-Zoume, Agbanhizoun, and Zogbodome, respectively. In northern Benin, sampling was performed in the two national parks (W Park and Pendjari Park). These sampling sites are illustrated in Figure-1.

### Materials

To achieve our goal, we used a stereo-microscope (Zeiss Stemi 2000) with a 60× resolution, a microscope with a 100× resolution, a Geographic Position System, 70° of ethanol, plastic bottles, pliers, a pencil, and adhesive papers, A4 papers.

#### Animal species and period of study

Ticks were collected from February to March 2017 on 9 wild animal species that were freshly slaughtered and visibly infested (Table-1). Sampling area selection was made randomly.

**Table-1:** The selected animal species.

Scientific name	Common name	Number of animals
*Thryonomys swinderianus*	Cane rat	14
*Felis silvestris*	Wildcat	1
*Lepus* spp.	Hare	26
*Cervus elaphus*	Doe	2
*Cricetomys gambianus*	Cricetoma	10
*Syncerus caffer planiceros*	Buffalo	7
*Kobus kob*	Buffon Cobe	2
*Tragelaphus scriptus*	Bushbuck	8
*Phacochoerus aethiopicus*	Warthog	3
Total		73

#### Ticks collection

Ticks were manually picked with forceps from each animal after they were slaughtered by hunters. These ticks were kept in different vials of 100 ml containing 70° of ethanol. One vial is used per animal species. At the end of each sampling, a tag made of A4 paper with the necessary information (date of collection, sampling area, and animal species sampled) was directly inserted into each vial before it was completely closed.

#### Ticks identification

Ticks identification was made at the laboratory based on the identification key as described by Walker *et al*. [19] using a binocular loupe. The genus and species of each tick examined were recorded.

### Statistical analysis

The average number of ticks was calculated for the different factors including localities, animal species, and tick species. The same parameters were calculated for localities, and animal species for each species of tick observed. Then these averages were tested using a Kruskal–Wallis test. Histograms were also constructed to highlight the proportion of different stages of tick development per animal species and localities. In addition, the proportion of each tick species was calculated by animal species and locality to assess the importance of its prevalence. Finally, to evaluate the effect of the factors studied on tick’s abundance, the data were adjusted to the Poisson model since the latter come from counts. All analyses were done with R Core Team version 3.2.2 software (Vienna, Austria).

## Results

### Comparison of average number of ticks according to factors levels

The average number of ticks varies from 3 to 6 between animal species, 3 to 7 between visited localities, and 2 to 5 between tick species (Table-1). However, these differences are statistically significant only for localities (Table-1). With regard to animal species, the highest average number of ticks is obtained on doe *(Cervus elaphus)* and Wildcat *(Felis silvestris)* in the locality of Adomougon (Table-1). Considering simultaneously localities and tick species, *Rm* has the highest average number in the locality of Mekrou (Table-2). Taking into account tick species and animal species, the parasite load of *Rm* and *Rhipicephalus* spp. is higher and Buffalo is the most infested animal (Table-3).

**Table-2:** Average number of ticks and standard error per factor.

Animal species			Localities			Ticks species		
		
Modalities	Avg.	SE	Modalities	Avg.	SE	Modalities	Avg.	SE
Wildcat	6.00^a^	-	Adomougon	7.75^a^	2.17	*Av*	5.00^a^	3.00
Cane rat	5.00^a^	0.89	Hounkpogon	4.11^ab^	0.48	*Bd*	2.00^a^	0.00
Doe	6.00^a^	-	Koncombri	4.22^ab^	0.88	*Rm*	5.33^a^	0.91
Buffalo	4.78^a^	1.09	Mekrou	4.57^ab^	1.11	*Bsp*	3.75^a^	0.75
Buffon cobe	2.50^a^	0.50	Porga	2.40^b^	0.24	*Hsp*	3.17^a^	0.31
Cricetoma	4.00^a^	0.55	Segbohoue	5.50^ab^	0.96	*Rsa*	5.57^a^	1.65
Bushbuck	3.63^a^	0.68	Tegon	4.00^ab^	1.14	*Rsp*	4.58^a^	0.57
Hare	5.50^a^	1.45	-			-		
Warthog	3.50^a^	0.65						
Prob.	0.636		0.075			0.191		

*Av=Amblyomma variegatum, Bd=Boophilus decoloratus, Rm=Rhipicephalus microplus, Bsp=Boophilus spp., Hsp=Hyalomma* spp*, Rsa=Rhipicephalus sanguineus, Rsp=Rhipicephalus* spp. Prob.=Probability related to the significance of the Kruskal–Wallis test at the 5% threshold, Avg.=Average, SE=Standard error. Averages with same letters within the same column are not significantly different at 5%

**Table-3:** Average number of ticks and standard error by location and tick species.

Localities	Statistics	Tick species						

		*Av*	*Bd*	*Rm*	*Bsp*	*Hsp*	*Rsa*	*Rsp*
Adomougon	Avg.	-	-	7	6	-	14	4
	SE	-	-	-	-	-	-	-
Hounkpogon	Avg.	-	-	3.5	-	3	4	6
	SE	-	-	0.5	-	0	1	0
Koncombri	Avg.	-	2	8	3	3	-	4.75
	SE	-	-	-	-	1	-	1.55
Mekrou	Avg.	2	2	8.5	3	4	-	4
	SE	-	-	1.5	-	-	-	-
Porga	Avg.	-	2	2.5	-	3	-	2
	SE	-	-	0.5	-	-	-	-
Segbohoue	Avg.	-	-	4	-	-	6	6
	SE	-	-	-	-	-	2	-
Tegon	Avg.	8	-	-	3	-	1	4
	SE	-	-	-	-	-	-	0
	Prob.	0.317	-	0.354	0.391	0.900	0.867	0.403

SE=Standard error, Prob.=Probability of the significance of the Kruskal–Wallis test at only 5%. Avg.=Average, *Av=Amblyomma variegatum, Bd=Boophilus decoloratus, Rm=Rhipicephalus microplus, Bsp=Boophilus* spp*., Hsp=Hyalomma* spp*., Rsa=Rhipicephalus sanguineus, Rsp=Rhipicephalus* spp.

### Variation in tick number by sex and development stage

**Figure-2:**
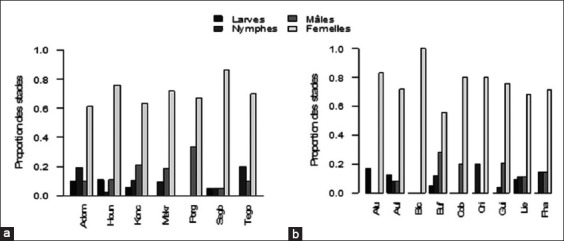
Proportions of the following tick stages (a) localities and (b) animal species. Localities: (Adom=Adomougon, Houn=Hounkpogon, Konc=Koncombri, Mekr=Mekrou, Porg=Porga, Segb=Segbohoue, and Tego=Tegon). Animals: (Alu=Wild cat, Aul=Cane rat, Bic=Doe, Buf=Buffalo, Cob=Buffon cobe, Cri=Cricetoma, Gui=Bushbuck, Lie=Hare, and Pha=Warthog).

Tick number collected within different localities and on different animal species was counted by sex and development stage. Overall, the results reveal that in all the localities and for all animal species considered, female ticks are the most prevalent (Figure-2a-b). Larvae or nymphs are less observed or almost absent in certain species such as the cricetoma (Gambia rat) and buffalo cobe. Table-4 shows that only *Rhipicephalus sanguineus* is found on doe (*C. elaphus*) whereas only *Rhipicephalus* spp. is found on wildcat *(F. silvestris)*. The other animal species are infested by several tick species; however, ticks belonging to *Boophilus* and *Rhipicephalus* genus are predominant.

**Table-4:** Average number of ticks and standard error per tick and animal species.

Animal species	Statistics	Tick species						

		*Av*	*Bd*	*Rm*	*Bsp*	*Hsp*	*Rsa*	*Rsp*
wildcat	Avg.	-	-	-	-	-	-	6
	SE	-	-	-	-	-	-	-
Cane rat	Avg.	8	-	-	-	3	4	5
	SE	-	-	-	-	-	-	1
Doe	Avg.	-	-	-	-	-	6	-
	SE	-	-	-	-	-	-	-
Buffalo	Avg.	2	2	7	-	3.5	-	9
	SE	-	0	2.08	-	0.5	-	-
Buffon cobe	Avg.	-	2	-	-	-	-	3
	SE	-	-	-	-	-	-	-
Cricetoma	Avg.	-	-	-	6	3	3	4
	SE	-	-	-	-	-	-	0
Bushbuck	Avg.	-	-	4	3	-	-	3.5
	SE	-	-	1.53	-	-	-	0.96
Hare	Avg.	-	-	5	3	-	6.5	-
	SE	-	-	1	-	-	2.9	-
Warthog	Avg.	-	-	-	3	3	-	5
	SE	-	-	-	-	1	-	-
	Prob.	0.317	-	0.2101	0.391	0.654	0.199	0.495

SE=Standard error, Prob.=Probability of the significance of the Kruskal–Wallis test at only 5%. Av*=Amblyomma variegatum, Bd=Rhipicephalus decoloratus, Rm=Rhipicephalus microplus, Bsp=Boophilus* spp*., Hsp=Hyalomma* spp*., Rsa=Rhipicephalus sanguineus, Rsp=Rhipicephalus* spp.

### Adjustment of the data to Poisson model

The deviance analysis table reveals that the abundance of ticks observed depends only on localities (p>0.05) (Table-5). However, the interactions between animal species and localities on the one hand and between animal and tick species on the other hand, although not significant, can influence the abundance of ticks as they reduce the residual deviance after their integration into the model.

**Table-5:** Full model deviance analysis table.

SSV	Df	Deviance	Resid. Df	Resid. Dev	Pr(>*Χ*)
Null	42	57.31			
Species	8	7.80	34	49.51	0.453
Localities	6	17.74	28	31.77	0.007**
Ticks	6	6.76	22	25.01	0.344
Species: Localities	7	9.56	15	15.45	0.214
Species: Ticks	9	11.02	6	4.43	0.274
Localities: Ticks	6	4.42	0	0	0.619
Species: Localities: Ticks	0	0	0	0	-

** Significant at the 5% threshold

## Discussion

Previous studies on domestic animal particularly cattle have shown the existence of for genus of ticks (*Amblyomma*, *Boophilus*, *Rhipicephalus*, and *Hyalomma*) in Benin. Meanwhile, these works revealed that September, October, November, June, July, and August are favorable to the proliferation of ticks and the months of February and March are periods of low abundance [20-22]. The results of our work on wild animals at Northern, central, and southern Benin also showed the presence of the four geniuses of ticks previously found on domestic animals. Morel [23] reported the presence of four kinds of ticks on wild animals in Benin including *Amblyomma, Boophilus Rhipicephalus*, and *Haemaphysalis*. The latter, specific to carnivores, was not identified in this study.

Among the ticks of Boophilus genius, we have identified three species, namely *Boophilus microplus, Boophilus decoloratus*, and *Boophilus* spp. [24] identified *Boophilus geigyi* in Benin on hartebeest and in Niger (north of W park) on hartebeest and roan. It has also been identified in Senegal on Luzarches hartebeest and then the bushbuck and warthog in the Niokolo-koba National Park. In the North East of Central Africa, the work of Thal [25] also mentioned the presence of this species on hartebeest and roan. Indeed, according to this author, it is the only *Boophilus* that could be found on wild ungulates in West Africa. However, although our samples were taken from ungulates such as *C. elaphus, Syncerus caffer planiceros, Kobus kob, Tragelaphus scrip tus*, and *Phacochoerus aethiopi cus*, this species has not been identified. Its absence could be linked to a mutation phenomenon leading to the appearance of *Rm*. This phenomenon was already described in East Africa where *Rm* replaced the other *Rhipicephalus* species [26]. The highest presence *Rm* in the hunting camp of the Mekrou and Koncombri can be explained by the fact that there are next to Burkina Faso. Moreover, the presence of water table next to W park hunting area in Popoman attract transhumant animals from Burkina-Faso and could explain the strong representation of *Rm* in the Mekrou area. Indeed, these camps are located at the Benin-Burkina border where there is a large movement of live cattle from Burkina to Benin through livestock trade. This is corroborated by recent findings that revealed the presence of this tick in Benin and Benin-Burkina Faso border [27].

However, the presence of *B. decoloratus* mentioned in this study does not confirm the results of Lamontellerie [28] who state that *B. decoloratus* is a tick of arid environments an only found on livestock and never on wild ungulates. Several authors have reported its presence on cattle in Benin [23,29]. Its presence on wild ungulates may be due to the possibility of being transported on long distances by the host from one environment to another as it development cycle is monophasic.

Altogether, two species of *Boophilus* were identified in our study. The undefined (*Boophilus* spp.) species may be originated from the different crossings that occur, nowadays, between *Boophilus* species [7].

One species of *Rhipicephalus (R. sanguineus)* and some undefined species (*Rhipicephalus* spp*.)* were identified*. R. sanguineus* is collected from doe (*C. elaphus)*, cricetoma (*Cricetomys gambianus*), hare (*Lepus* spp.), and cane rat (*Thryonomys swinder ianus*) in areas located in Central and Southern Benin. However, *Rhipicephalus* spp. is present in all the prospected localities. The absence of *R. sanguineus* in this study in the parks may be related to the prohibition of hunting that would have limited dog access to the parks. In fact, illegal hunters are often accompanied by their dogs, which constitute the main host of *R. sanguineus* in the tropics and subtropics as reported by Fahmy *et al*. [30]. A high probability of dissemination of this tick on the natural route by these dogs and thus an infestation of wild animals is possible. This has been proven by the work of Smith *et al*. [31].

*Amblyomma* tick is widespread in Africa, and its hosts are domestic and wild ruminants such as buffaloes, cattle, sheep, and goats [32]. However, they are able to infest others animals species. This is the case in our study where *Amblyomma variegatum* was found on cane rats (*T. swinderianus*).

The buffalo hosts two specific ticks, *Amblyomma splendidum* and *Rhipicephalus cliffordi*; both species were identified in Ivory Coast, and *A. splendidum* only in central Benin [23].

None was identified in western, central, and eastern Burkina-Faso although the number of buffalo one by the country [23].

Similarly, Barre [33] found this tick, at the adult stage, on Caribbean and Guadeloupe dog’s, where it was introduced 150 years ago. Our results confirm the great variability of hosts susceptible to be infested by the ticks of the *Amblyomma* genus.

*Hyalomma* spp. populations have been identified on *C. gambianus*, *T. swinderianus, S. caffer*, and *P. aethiopicus*. This demonstrates its ability to infest a diversity of hosts. These results are in concordance with those obtained by Morel [23]. Indeed, *Hyalomma* ticks are generally found on wild ungulates and rodents. The same author reports the presence of *Hyalomma nitidum* in Central African Republic where it was collected on Buffalo, Antelope, Buffalo Cob, and warthog. It is also known in Senegal and Benin.

Of all these species, it should be noted that the proportion of nymph and larva is very low or almost absent. Indeed, previous work carried in the same areas and during the same month’s revealed low abundance of nymph and larva [34]. This observation can be explained by several factors such as the height of the animals considered, targeted animals are old, and old animals are big in size what make does not favor nymph and larva to attach to them; the climate: February and March are hot and larva and nymph are more sensitive to heat than adults. Moreover, this period is the most favorable for the availability of wildfowl, and illegal hunters use bushfires to find wildfowl. As a result, these bushfires, kill immature ticks hidden in the environment. According to Verdonck **[**35], immature survival is very dependent on humidity during the hot season; the abundance of *Rm*, for example, begins to decrease at the end of the rainy season. Likewise, tick activity varies according to species and climatic conditions [36]. However, the massive presence of ticks does not always correspond to the rainiest months. Farougou *et al*. [22] showed a low correlation between rainfall and number of ticks collected. This result can be explained by the fact that some ticks appear before the rainy season. The finding may also be related to genus and stage of development that persist in moisture and sometimes other sources of moisture at the vegetation level. Similarly, Fahmy *et al*. [30] showed that *Rm* can grow in warm and humid regions, *B. decoloratus* in dry and cold areas, and *Boophilus annulatus* can survive in areas suitable both for *Rm* and *B. decoloratus*. This fact has also been confirmed by several researchers [37,38]. Madder *et al*. [27] and Muhimuzi *et al*. [39] also reported that *B. annulatus* and *B. geigyi* might share the same habitats associated with woods and forests and favor similar environmental requirements as well.

In summary, during this cross-sectional study on wild animals in Benin, seven species of ticks were identified including *Rm* on buffalo, the bushbuck, and the hare. Although the number of animals surveyed and the number of ticks taken are low for specific reasons related to the low parasite load of the animals encountered, the reluctance of some illegal hunters and the limited number of legal hunters with a hunting license at the level of national parks. 24 tick species have been recorded on wild animals in Upper Volta [23]; In the Democratic Republic of Congo, three species of ticks were identified on natural grasslands, including *Rhipicephalus* *appendiculatus, B. decoloratus*, and *Haemaphysalis Leachi Leachi* [39]. The lower number of tick collected in our case is due to the reluctance of some hunters to allow ticks collection from their animals. Moreover, the number of hunters that can be survey is also limited as few hunters own a hunting license.

## Conclusion

This study shows that *Rm* is established in the wildlife of Benin. Its presence confirms the reservoir role and maintenance that wildlife plays in the spread of parasites. Wildlife is, therefore, an important risk factor that should not be neglected in the epidemiological surveillance and tick control strategies particularly *Rm* in Benin and the West African sub-region. Thus, it is important to involve in the programs of protection of the faunistic reserves the control of ticks in particular and a sanitary management of wildlife in general.

## Authors’ Contributions

KJA; BS, GAM; YA, and SBA have participated in developing the protocol, the sample of ticks and in drafting the manuscript. REY participated in the identification of ticks and the development of the database. CA contributed to the translation of the manuscript. AKIY; SF and AIS supervised the analysis of the statistical results and the correction of the manuscript. All authors have read and approved the final manuscript.
